# Magnetic immobilization of a quorum sensing signal hydrolase, AiiA

**DOI:** 10.1002/mbo3.797

**Published:** 2019-02-14

**Authors:** Lin Wang, Haixing Xu, Zewen Liu, Taolei Sun, Chengqing Yuan, Ying Yang, Junhui Guo, Hao Xie

**Affiliations:** ^1^ School of Chemistry, Chemical Engineering, and Life Science Wuhan University of Technology Wuhan China; ^2^ School of Energy and Power Engineering Wuhan University of Technology Wuhan China; ^3^ Institute for Science and Technology in Medicine Keele University Staffordshire UK

**Keywords:** AiiA, hydrolase, magnetic immobilization, MagR, quorum sensing signal

## Abstract

Magnetic immobilization of quorum sensing (QS) signal hydrolases provides a convenient solution for quenching QS process that is essential for bacterial biofilm formation and antimicrobial resistance. In the present study, a QS signal hydrolase, AiiA, was fused with a magnetic protein, MagR, and expressed in *Escherichia coli*. Magnetic immobilization of AiiA was achieved on Fe_3_O_4_‐SiO_2_ iron beads and was confirmed via SDS‐PAGE, zeta potential measurement, FTIR spectrometry, and SEM analysis. The magnetic immobilized AiiA exhibited activity in degrading the quorum sensing signal, C6‐HSL. This study opens a new avenue to actively immobilize enzymes via magnetic interaction and quench quorum sensing.

## 1 INTRODUCTION

Quorum sensing (QS) is a bacterial type of cell–cell communication by which bacteria regulates the expression of specific genes involved in biofilm formation, adhesion, antibiotic synthesis, virulence, swarming, bioluminescence, mating, and tissue damage (Bassler, 2002; Davies, Parsek, Pearson, Iglewski, & Costerton, 1998). For example, bacteria sense and regulate the growth of bacterial group via QS effects and form biofilm on implant surface (VanEpps & Younger, 2016). It contributes to the antimicrobial resistance that leads to the failure of anti‐infection treatment after implant surgery since biofilm not only retards the diffusion of antibiotics, but also prevents pathogenic bacteria from attacking of immune cells. Therefore, bacterial QS is recognized as an important target for anti‐infective immunotherapy (Kaufmann, Park, & Janda, 2008).

Bacterial QS process can be quenched by decreasing QS signal levels with enzymes, antibodies, as well as scavenging or processing by competing organisms (Amara, Krom, Kaufmann, & Meijler, 2011). N‐acyl‐L‐homoserine lactonases (AHLs) are among the most widely studied QS molecules in gram‐negative bacteria (Fuqua, Parsek, & Greenberg, 2001; Teplitski, Mathesius, & Rumbaugh, 2011). AHL‐degrading enzymes have been found to have significant commercial value as a means to control cell‐to‐cell signaling (Dobretsov, Teplitski, & Paul, 2009; Dong & Zhang, 2005; Teasdale, Liu, Wallace, Akhlaghi, & Rowley, 2009). For example, AiiA is an AHL hydrolase (also called AHL, E.C.3.1.1.81) that catalyzes the hydrolysis of the AHL ring, which makes AiiA a strong candidate for developing bio‐decontaminating agent in industrial and environmental setting as well as in medical use (Bai, Han, Chen, & Zhang, 2008; Dong, Xu, Li, & Zhang, 2000; Ponce, Martins, Araujo, Mantovani, & Vanetti, 2012).

Practically, inhibition of QS is often required on specific surfaces such as implant surface. This can be achieved by immobilizing enzymes to degrade or modify QS signals in a specific area. Generally, enzyme can be immobilized with chemical or physical means by modifying enzymes or supports/carriers to allow specific interactions between each other, by using bi‐ or multifunctional coupling agents to bridge enzymes with supports/carriers, or by genetically modifying enzymes with fusion tags to provide the enzymes with binding affinity to supports/carriers (Tischer & Kasche, 1999). Various proteins or domains with affinity to matrix have been used as fusion tags for immobilizing enzymes. Representatives include polyhistidine tag (Chen, Brash, & Funk, 1993), Strep II tag (Smyth, Odenthal, Merkl, & Paulsson, 2000), MBP tag (Lichty, Malecki, Agnew, Michelson‐Horowitz, & Tan, 2005), and GST tag (Scheich, Sievert, & Bussow, 2003), which facilitate the purification of many proteins. However, interactions between conventional fusion tags rely on pH, salinity, and ionic strength of the environment, which may not be suited to the activity of tagged enzymes. The use of magnetic protein MagR as fusion tag has been recently reported for actively immobilizing GFP and lipase (Jiang, Zhang, Wang, Zhang, & Liu, 2017). The magnetic interactions between MagR and supports imply that the MagR‐based immobilization is less affected by chemical environments. Therefore, the enzyme can be immobilized over a broad range of environmental conditions suiting its activity.

In the present study, the magnetic protein MagR was used as a fusion tag for magnetic immobilization of the QS signal hydrolases, AiiA. The recombinant protein AiiA‐MagR was constructed and overexpressed in *Escherichia coli* host. Magnetic immobilization of AiiA‐MagR was realized on Fe_3_O_4_‐SiO_2_ iron beads (IBs) and acquired AiiA‐MagR‐coated IBs (IB@[AiiA‐MagR]), which was characterized by techniques including SEM and FTIR. The enzymatic activity of IB@[AiiA‐MagR] in degrading AHLs was analyzed.

## 2 MATERIALS AND METHODS

### 2.1 Molecular biology manipulation

The quorum sensing signal hydrolase AiiA was expressed as MagR fusions. The DNA segments encoding MagR (GenBank Accession NP_573062) and AiiA (GenBank Accession AGA83379) were optimized for prokaryotic expression and synthesized by Bio Translation Lab. The coding sequence of AiiA was cloned into the multiple cloning sites of plasmid pET28a containing MagR, resulting in the AiiA protein placing at N‐terminus of MagR. The resultant vector was named as pET28a[AiiA‐MagR] with the sequence was confirmed by DNA sequencing.

### 2.2 Expression of AiiA‐MagR fusion protein

Expression of AiiA‐MagR fusion protein was based on Jiang et al. (2017). Colonies of *E. coli* BL21 (DE3) harboring plasmid pET28a[AiiA‐MagR] were inoculated into Luria‐Bertani medium containing 50 μg/ml kanamycin and shaken at 37°C overnight. The cell suspension was inoculated into LB medium and followed by shaking at 37°C until the value of OD600 reached 0.5–0.6. Protein expression was initiated by supplying with 0.2 mM of IPTG and was continuously shaken at 20°C for 10–14 hr. Cells were then harvested by centrifugation at 6,000 *g* for 10 min and stored at −80°C.

### 2.3 Magnetic immobilization of AiiA‐MagR

The cell pellet (0.5–1 g) with expressed AiiA‐MagR was resuspended in 5–10 ml of ice‐cold TBS buffer (20 mM Tris, 150 mM NaCl, pH 7.5) containing 0.2% (w/v) lysozyme and disrupted two times by continuous flow of high‐pressure freezing cells broken TSO.75 KW (Constant Systems, UK). The cell lysate was centrifuged at 10,000 *g* for 30 min at 4°C. The supernatant was collected and mixed with 10 mg/ml Fe_3_O_4_‐SiO_2_ iron beads (IBs, BeaverBeads) at a volume ratio of 5:1. The mixture was agitated for 30 min to allow complete interactions between AiiA‐MagR and IBs. The resultant precipitation was harvested by centrifugation and washed three times with TBS buffer to remove unbound proteins. The resultant IBs with coated AiiA‐MagR was labeled as IB@[AiiA‐MagR]. The immobilization efficiency of AiiA‐MagR on IBs was analyzed by heating IB@[AiiA‐MagR] at 90°C for 10 min. The proteins were then analyzed by using SDS‐PAGE. The amount of protein was determined by using BCA Protein Assay Kit with bovine serum albumin (BSA) as the standard.

### 2.4 Characterization of IB@[AiiA‐MagR]

The morphology of IB@[AiiA‐MagR] was imaged by using a S4800 (Hitachi Company) scanning electron microscope (SEM), fitted with a field‐emission source operating at 5 kV. The zeta potential was measured using a Zetasizer Nano‐ZS90 (Malvern, UK). The FTIR spectra of pellets were collected from 4,000 to 400 cm^−1^, at a resolution of 4 cm^−1^ with 64 scans by using a Nexus (Thermo Fisher Scientific) Fourier transform infrared (FTIR) spectrometer.

### 2.5 Enzymatic assay of IB@[AiiA‐MagR]

The AHL activity of IB@[AiiA‐MagR] was assessed with a C6‐HSL diffusion assay (Jiang et al., 2017) by using *Chromobacterium violaceum* CV026 (kindly provided by Professor Zhu Hu, China University of Petroleum, China) as the reporter bacterial strain. Since CV026 produces purple pigment only in the presence of acylated homoserine lactone (AHL), the degradation of AHL will significantly reduce the color. Briefly, a certain amount of C6‐HSL (0.2–0.8 nmole) in 50 mM phosphate buffer (pH 8.0) was mixed with 1 mg of IB@[AiiA‐MagR]. Degradation of C6‐HSL was initiated by shaking the reaction mixture at 220 rpm for 60 min at 37°C. The reaction mixtures were transferred into one of two wells (5 mm in diameter) on an agar plate that was plated with 400 μl of *C. violaceum *CV026. The control group (IB‐treated C6‐HSL) was transferred into the other well in the same plate. The plate was then incubated at 28°C. The AHL degradation was exhibited by the reduction or abolishment of purple pigments after 24 hr of incubation (Ab Ghani, Norizan, Chan, Yin, & Chan, 2014). For indexing the degradation efficiency, the density and area of purple color were measured with ImageJ and compared with the control group.

The AHL activity of IB@[AiiA‐MagR] was also quantitatively assessed through a colorimetrical assay of the purple pigment violacein (Mukherji, Varshney, Panigrahi, Suresh, & Prabhune, 2014). Aliquots of 20 μl phosphate buffer (pH 7.0) containing 1 mmol AHL (C6‐HSL) and 1 mg IB@[AiiA‐MagR] were incubated at 35°C for enzymatic degradation of C6‐HSL. The reaction aliquot was taken hourly and diluted in 5 ml LB broth containing 100 μl of overnight culture of CV026 to terminate reaction, followed by incubating at 30°C for 16–18 hr. The violacein produced was extracted from the culture broth by dissolving the pigment in 1 ml DMSO. After removing the cell mass by centrifugation, the purple pigment production was quantitatively estimated by measuring the absorbance at 570 nm.

### 2.6 Biofilm assay

The biofilm formation was quantified using the crystal violet staining method (Cao, Yang, Uche, Hart, & Li, 2018; Vinoj, Pati, Sonawane, & Vaseeharan, 2015). Different amount of IB@[AiiA‐MagR] (0, 0.025, 0.05, 0.1, 0.2, 0.4 mg) was added into 24‐well suspension culture plates. The *P. aeruginosa* PA01 bacterial suspension at an OD_600_ of 0.05 (1 ml/well) and one piece of sterile catheters (1 × 0.5 cm^2^) were added into each well. Plates were then incubated at 37°C for 24 hr. The catheter pieces were removed from the plate and washed three times using sterilized PBS to remove cell medium and nonadhered bacteria. The plates were dried at ambient temperature for 20 min and then stained with 0.1% crystal violet for 10 min. Residual solution was then removed, and samples were washed four times using sterilized PBS. Plates were air‐dried before adding 1 ml 30% acetic acid to each well and recording spectrophotometric absorbance at 590 nm.

## 3 RESULTS AND DISCUSSION

### 3.1 Construction and expression of MagR‐Tagged AiiA

In the present study, a plasmid harboring the DNA sequence encoding segments of AiiA and MagR was constructed to facilitate expression of MagR‐tagged AiiA in *E. coli *host. The sequence of protein AiiA was based on AHL from *Bacillus cereus* isolated from striped catfish (*Pangasianodon hypophthalmus*) pond (Tinh, Dung, Trung, & Thuy, 2013). The sequence of protein MagR was based on magnetic receptor, isoform A from *Drosophila melanogaster* (Cyranoski, 2015). The resultant recombinant protein was named as AiiA‐MagR with the MagR protein placing at C‐terminus of AiiA.

**Figure 1:**
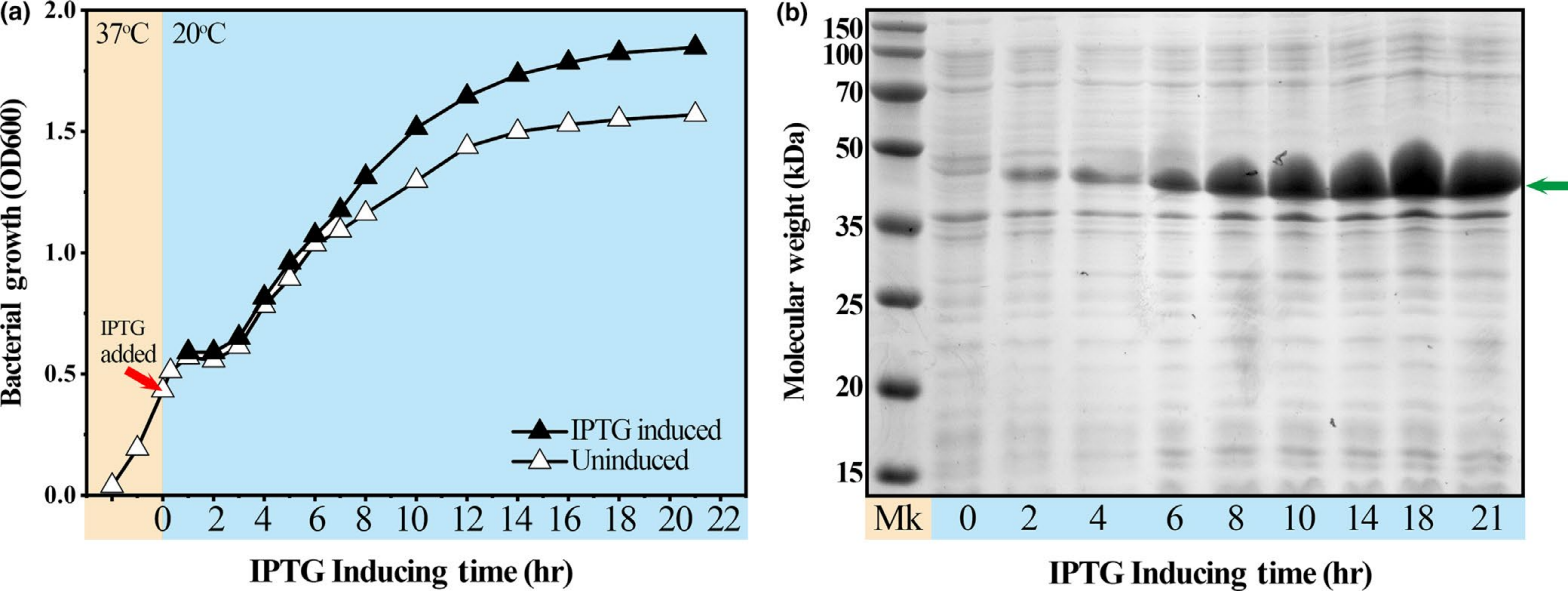
Expression of recombinant AiiA‐MagR. Panel (a), IPTG‐induced growth curve of bacterial cells containing expression vector pET28a[AiiA‐MagR]. Panel (b), SDS‐PAGE analysis of AiiA‐MagR expression in IPTG‐induced cells. Lane Mk, molecular weight markers; lanes 0, 2, 6, 8, 10, 14, 18, and 21 were loaded with lysates of cells upon IPTG induction of 0, 2, 6, 8, 10, 14, 18, and 21 hr

Expression of recombinant AiiA‐MagR was initiated by supplying the bacterial culture with IPTG. Upon IPTG induction, *E. coli* containing AiiA‐MagR expression vector exhibited elevated growth than of uninduced *E. coli *(Figure 1, Panel a). Expression of AiiA‐MagR with theoretic molecular weight of 44.4 kDa was observed by comparing the protein expression in whole cell extracts between IPTG‐induced *E. coli* and uninduced cells (Figure 1, Panel b). The expression level of AiiA‐MagR increased with the increase in IPTG inducing time. After 14 hr of IPTG induction, it almost reached the highest level of AiiA‐MagR expression as well as the growth of bacteria. It implied that 14 hr of IPTG induction was sufficient to produce bacteria with expressed recombinant AiiA‐MagR. Longer time of IPTG induction would not be advantageous to protein production.

### 3.2 Magnetic immobilization of recombinant AiiA‐MagR

**Figure 2:**
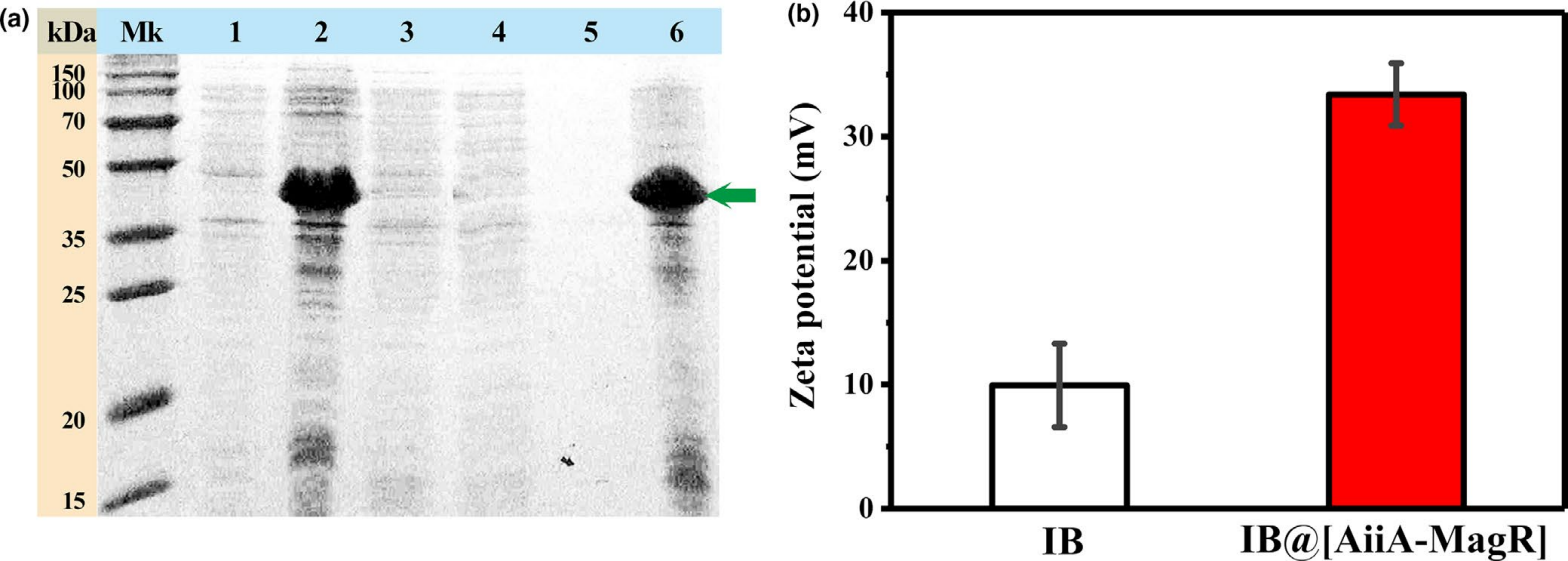
Interaction between IB and AiiA‐MagR. Panel (a), SDS‐PAGE analysis of AiiA‐MagR binding on IB. Lane Mk, molecular weight markers; lane 1, lysate of uninduced cells; lanes 2 and 3, lysate of IPTG‐induced cells before (lane 2) and after interaction with IB; lanes 4 and 5, fraction from wash step to remove weakly bound proteins from IB; lane 6, proteins bound on IB. Panel (b), surface charge (zeta potential) of IB samples before (indicated as IB) and after (indicated as IB@[AiiA‐MagR]) interaction with AiiA‐MagR

The expressed AiiA‐MagR was subjected to interact with Fe_3_O_4_ iron beads (IBs). The binding of AiiA‐MagR to IBs was evidenced with SDS‐PAGE analysis (Figure 2, Panel a). It was observed that significant amount of impurity was removed by wash. The binding capacity (immobilization efficiency) of IB to AiiA‐MagR was determined as 5.1 ± 0.3 mg/g. The surface charge (zeta potential) of IBs was analyzed before and after interaction with AiiA‐MagR (Figure 2, Panel b). The increase in the zeta potential was observed from 10 mV to about 35 mV, indicating that the stability of IBs was improved upon interacting with AiiA‐MagR.

**Figure 3:**
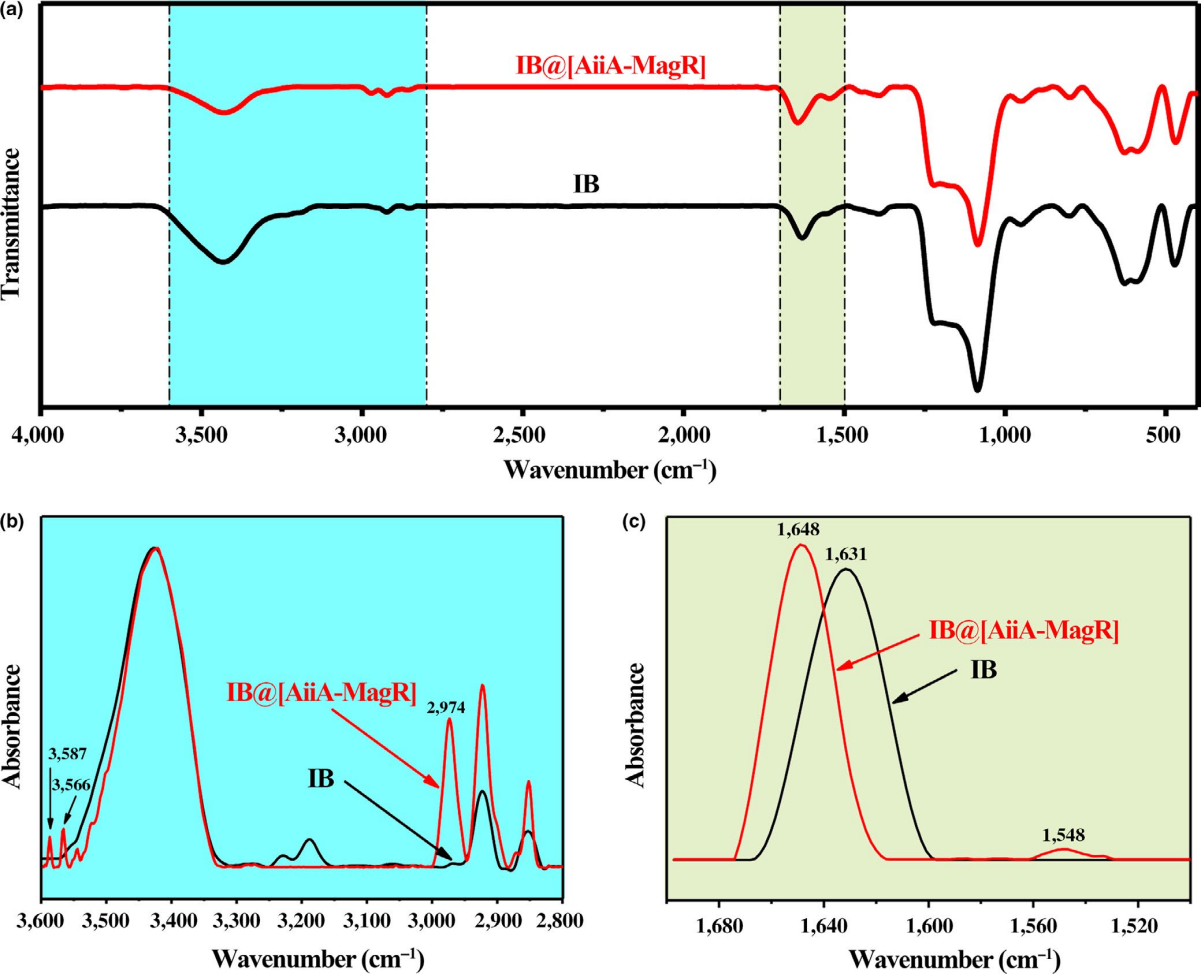
FTIR spectra of IB samples before (indicated as IB) and after (indicated as IB@[AiiA‐MagR]) interaction with AiiA‐MagR. (a), the transmittance spectra; (b) and (c), the absorbance spectra

Interaction between IBs and IB@[AiiA‐MagR] was analyzed with FTIR spectrometry (Figure 3). The difference of spectrum between IBs and IB@[AiiA‐MagR] was observed in the range of 2,800–3,600 cm^−1^ and 1,500–1,700 cm^−1^ (Figure 3, Panels a–c). After interaction with AiiA‐MagR, two characteristic peaks were detected at 3,566 cm^−1^ and 3,587 cm^−1^ that were assigned to stretching vibrations of primary amines as well as the band at 2,974 cm^−1^ was identified as secondary amine band. The characteristic bands in amide I region between 1,500 and 1,700 cm^−1^ correspond to the secondary structure of the polypeptide backbone. After binding with AiiA‐MagR, there was a red shift of the absorbance peak from 1.630 cm^−1^ to 1,648 cm^−1^ as well as the appearance of the characteristic peak at 1,548 cm^−1^ that was assigned to the bending vibration of amine. These data supported that IBs were coated with AiiA‐MagR.

**Figure 4:**
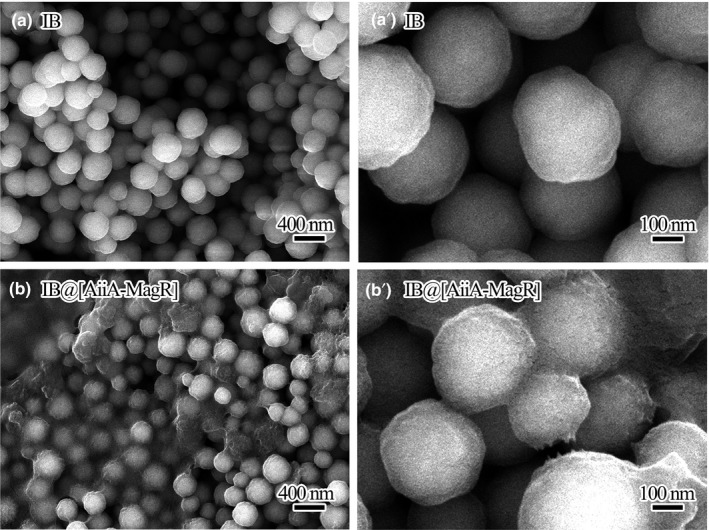
SEM analysis of IB samples before (Panels a and a′) and after (Panels b and b′) interaction with AiiA‐MagR

The morphological changes of IB after coating with AiiA‐MagR protein were further investigated with SEM (Figure 4). A coating layer was observed on IB@[AiiA‐MagR], which made the surface of IB@[AiiA‐MagR] rougher than that of IB. Therefore, it can be concluded that the magnetic immobilization of AiiA‐MagR on IB has been successfully achieved.

### 3.3 Enzymatic activity of IB@[AiiA‐MagR]

**Figure 5:**
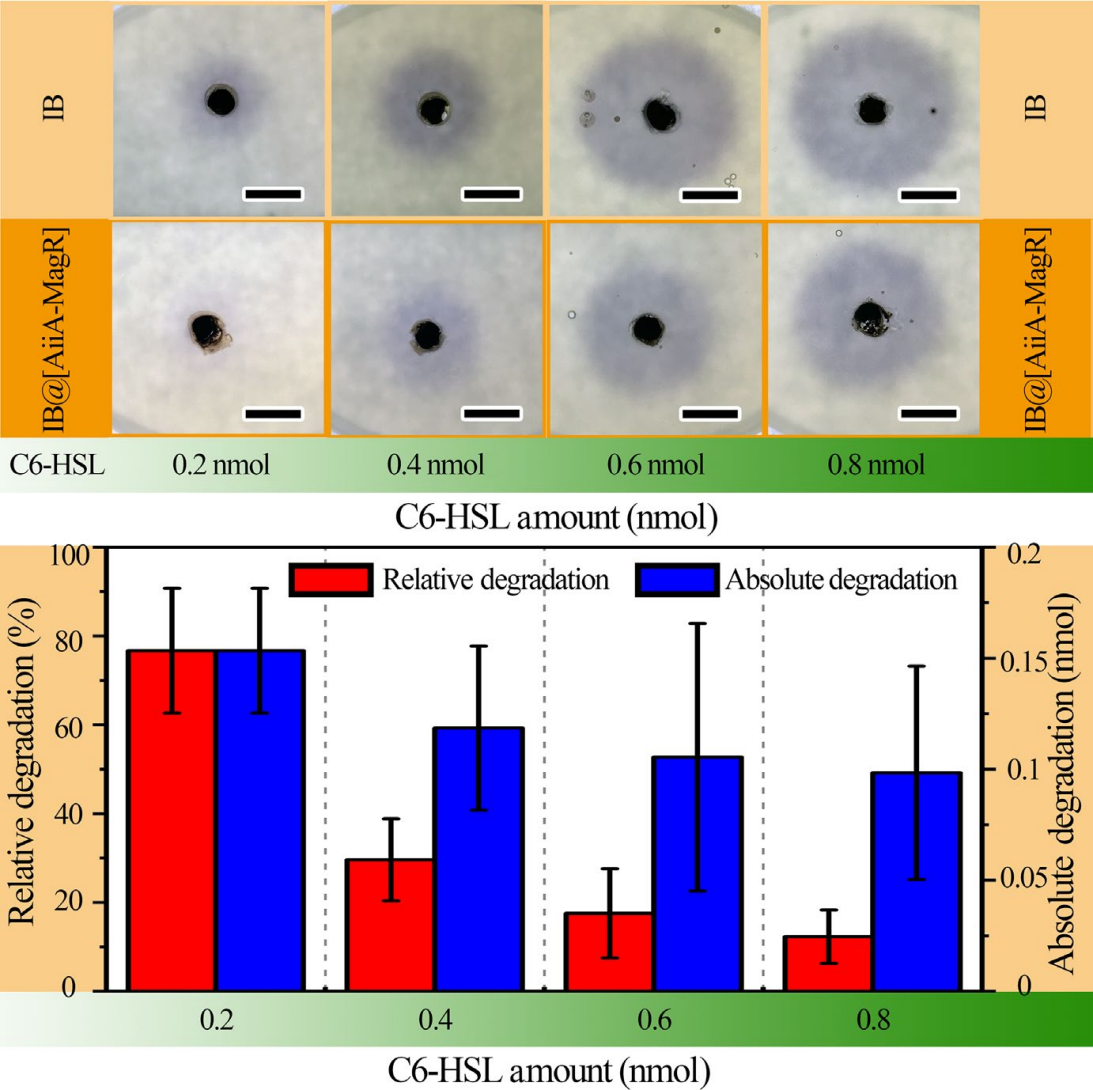
Enzymatic activity of IB@[AiiA‐MagR]. Up panel, pigment production by c. violaceum CV026 under function of different amount of C6‐HSL that were pretreated with IB or IB@[AiiA‐MagR]. Bottom panel, the quantitative analysis of the relative and absolute degradation of C6‐HSL based on pigment production that was indicated by area and density purple color

Enzymatic activity of magnetically immobilized AiiA‐MagR in degrading C6‐HSL was evaluated with C6‐HSL diffusion assay (Figure 5). Increase in area and density of purple color was observed when the amount of C6‐HSL was increased in the absence of AiiA‐MagR (Figure 5, up panel, IB group), indicating pigment production by *Chromobacterium violaceum *CV026 was related the amount of C6‐HSL in environment. In the presence of IB@[AiiA‐MagR] (Figure 5, up panel, IB@[AiiA‐MagR] group), decreases in area and density of purple color (pigment production) were observed, indicating the degradation of C6‐HSL. IB@[AiiA‐MagR] exhibited higher relative degradation efficiency in degrading small amount of C6‐HSL than large amount of C6‐HSL. However, the absolute degradation of C6‐HSL by IB@[AiiA‐MagR] did not exhibit significant difference between different C6‐HSL groups. It was estimated that 0.1–0.15 nmole C6‐HSL could be hydrolyzed by 0.11 nmole AiiA‐MagR (1 mg IB@[AiiA‐MagR]) in 60 min at 37°C.

**Figure 6:**
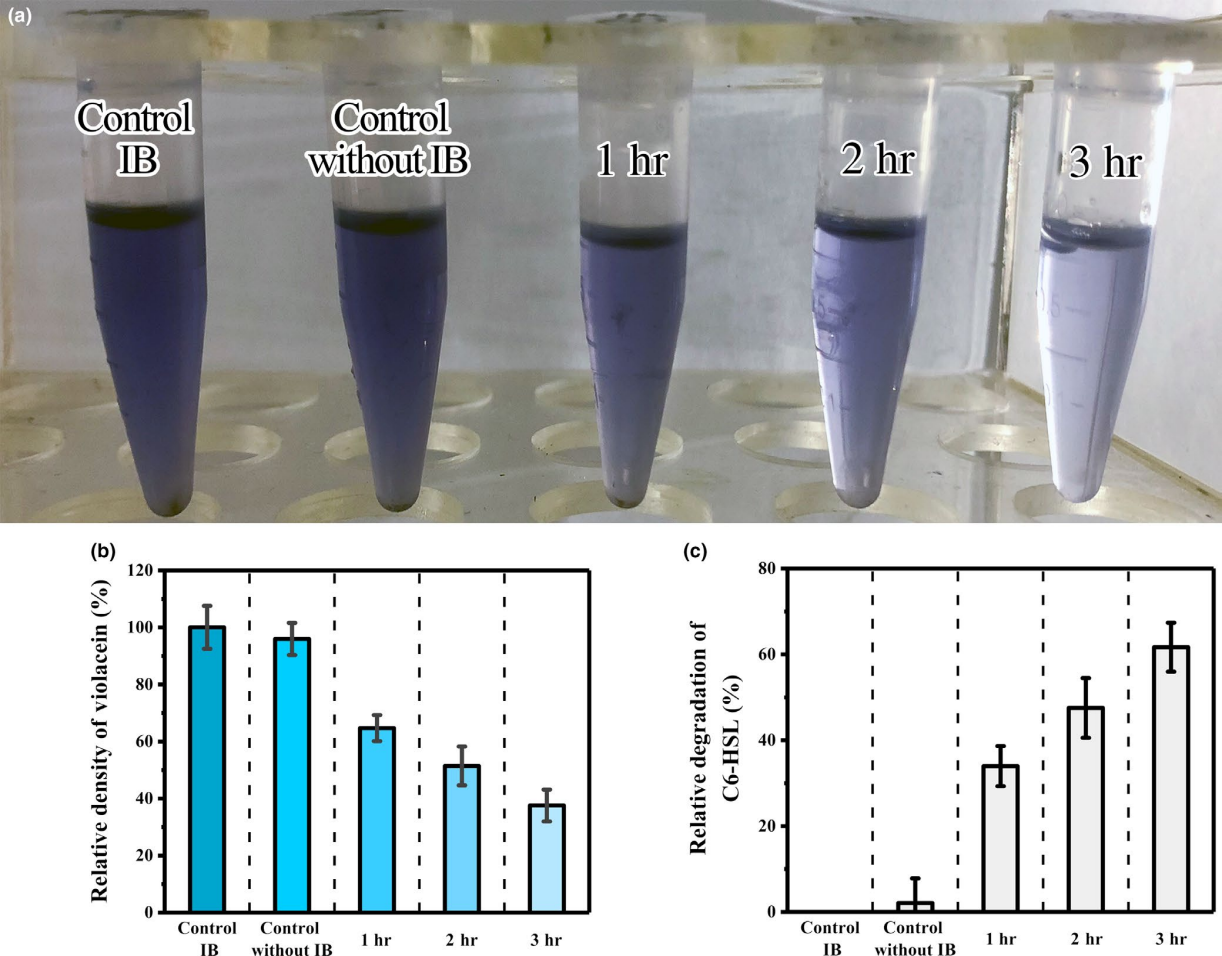
Degradation of C6‐HSL by IB@[AiiA‐MagR]. Panel a, the pigment violacein production by CV026; Panel b, the relative degradation of violacein; Panel c, the relative degradation of C6‐HSL. Control IB, samples with IB treatment; Control without IB, sample without IB treatment; 1, 2, and 3 hr are samples with IB@[AiiA‐MagR] treatment for 1, 2, and 3 hr

The AHL hydrolase activity of IB@[AiiA‐MagR] was also estimated by quantitatively analyzing the pigment violacein production by *C. violaceum* CV026 in liquid culture. After 3‐hr degradation of C6‐HSL by IB@[AiiA‐MagR], it was observed the density of purple color of violacein was reduced in liquid culture of *C. violaceum* CV026 after supplying with the reaction mixture and incubation overnight (Figure 6, Panels a, b). The longer the degradation time of C6‐HSL by IB@[AiiA‐MagR], the lighter the color of purple color of violacein, indicating the amount of C6‐HSL was decreased after interacting with IB@[AiiA‐MagR] (Figure 6, Panel c). During the testing time of 3 hr, the linearized degradation was observed, indicating long‐time treatment by IB@[AiiA‐MagR] could facilitate the hydrolysis of C6‐HSL. These results demonstrated the enzymatic activity of IB@[AiiA‐MagR] in depredating the QS signal, AHL.

### 3.4 Inhibition of Biofilm Formation by IB@[AiiA‐MagR]

**Figure 7:**
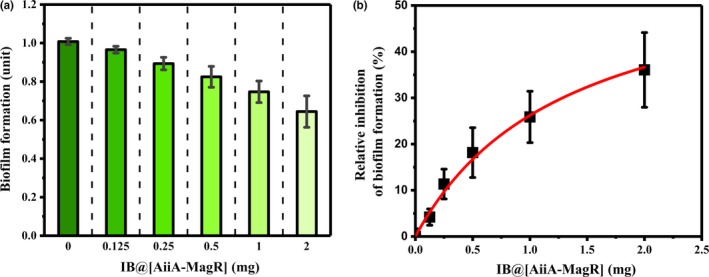
Biofilm formation of *P. aeruginosa PA01* on sterile catheters. Panel a, the absolute biofilm formation on 1.0 × 0.5 cm^2^ sterile catheter under the function of different amount of IB@[AiiA‐MagR]. Panel b, the inhibition of biofilm formation by IB@[AiiA‐MagR]

The biofilm formation of *P. aeruginosa PA01* on 1.0 × 0.5 cm^2^ sterile catheters was investigated under the function of IB@[AiiA‐MagR]. It was observed that increasing the amount of IB@[AiiA‐MagR] significantly reduced the biofilm formation (Figure 7, Panel a). It was also observed that the relative inhibition of biofilm by IB@[AiiA‐MagR] on sterile catheters follows the Michelis–Menton kinetics (Figure 7, Panel b), which calculated the maximum relative inhibition of biofilm was 61.0 (± 10.8)%, and half of the maximum relative inhibition of biofilm could be reached at the amount of 1.33 (± 0.36) mg of IB@[AiiA‐MagR], with the R square of 0.985. These results demonstrated the function of IB@[AiiA‐MagR] in inhibiting the biofilm formation of bacteria.

## 4 CONCLUSION

Enzyme immobilization is important in modern biotechnology and with applications in broad areas such as industrial production of antibiotics and beverages, diagnosis and treatment of diseases, and treatment of sewage and industrial effluents. Enzyme immobilization can be achieved with various techniques such as affinity‐tag binding, adsorption on supports, entrapment, cross‐linkage, and covalent bond. With gene fusion technology, a segment of peptide as a fusion tag can be engineered into a target protein at the genetic level and provide it with biochemical properties of the imported fusion tag. In the present study, a magnetic protein, MagR, as a fusion tag was placed at C‐terminus of a quorum sensing signal hydrolase, AiiA. The resultant recombinant protein AiiA‐MagR was successfully expressed in the *E. coli *host. The interaction between AiiA‐MagR and Fe_3_O_4_‐SiO_2_ iron beads (IBs) was observed and confirmed by SDS‐PAGE, zeta potential measurement, FTIR spectrometry, and SEM analysis. The binding capacity of IB to AiiA‐MagR was 5.1 ± 0.3 mg protein per gram IBs. The AiiA‐MagR coating on IBs remained activity in degrading the quorum sensing signal, C6‐HSL. The activity of AiiA‐MagR coating on IBs was detected in inhibiting biofilm formation of *P. aeruginosa PA01 *on sterile catheters. This study proved that the magnetic protein MagR could be used as a magnetic fusion tag to AiiA and facilitated the active magnetic immobilization of this QS signal hydrolase as well as reduced biofilm formation of bacteria. This magnetic immobilized AiiA‐MagR can be further used for quenching QS and inhibiting biofilm formation in various environments.

## CONFLICT OF INTEREST

The authors declare no financial or commercial conflict of interest.

## AUTHORS CONTRIBUTION

L. Wang and H. Xu conceived and designed experiments. L. Wang and Z. Liu performed experiments and collected data. L. Wang and H. Xie analyzed the data, prepared figures, and wrote the article. C. Yuan, Y. Yang, T. Sun, and J. Guo reviewed and edited the manuscript. All authors read and approved the manuscript.

## ETHICS STATEMENT

None required.

## Data Availability

The data will be available on request from the corresponding author.
